# Comparison of low-level laser therapy versus neuromuscular electrical nerve stimulation at hemiplegic shoulder pain and upper extremity functions

**DOI:** 10.1007/s10103-025-04305-1

**Published:** 2025-01-24

**Authors:** Pınar Özge Başaran, Dilek Eker Büyükşireci

**Affiliations:** Department of Physical Medicine and Rehabilitation, Hitit University Erol Olçok Education and Research Hospital, Çorum, Turkey

**Keywords:** Hemiplegic shoulder pain, Low-level laser therapy, Neuromuscular electrical nerve stimulation, Stroke, Upper extremity functions

## Abstract

This study aimed to assess and compare the effectiveness of adding low-level laser therapy (LLLT) and neuromuscular electrical nerve stimulation (NMES) to conventional physical therapy exercises, for stroke patients with hemiplegic shoulder pain (HSP). Seventy-five stroke patients with shoulder pain were included in this prospective randomized controlled study. Participants were divided into three groups. All patients underwent a multidisciplinary rehabilitation program five days a week for four weeks for a total of twenty sessions with classical physical therapy exercises. In addition, Group 1 received LLLT for three days a week for four weeks. Group 2 received NMES for twenty minutes for five days a week for four weeks. Group 3 control group received classical physical therapy exercises. Brunnstrom (BRS) upper extremity, BRS Hand, Barthel index, Shoulder Pain and Disability Index (SPADI), Fugl Meyer, Modified Ashworth Scale (MAS) and visual analog scale (VAS) were assessed, prior to the treatment and at the end of four weeks. After treatment, statistically significant improvements were found in BRS upper extremity, BRS Hand, Barthel index, SPADI, Fugl Meyer and VAS in all three groups (all *p* < 0.005). When the groups were compared, significant improvements in Bartel, SPADI and VAS in the LLLT and NMES groups than the control group(all *p* < 0.005), however the LLLT and NMES groups were statistically similar. LLLT, NMES and conventional exercise therapy have demonstrated efficacy in treating HSP and improving upper extremity functions and disability. Laser and NMES were more effective while the effects of laser and NMES were similar.

## Introduction

Cerebrovascular events, affecting approximately nine million people worldwide, have become a significant cause of morbidity and mortality, particularly as the human lifespan increases [1]. With the advent of effective treatments in the acute phase, expectations regarding prognosis have improved. However, secondary complications frequently arise post-stroke, significantly disrupting the rehabilitation process. The upper extremity is more commonly affected than the lower extremity, with recovery being slower and more challenging. Most functional impairments related to the upper extremity involve shoulder problems, primarily due to impaired shoulder biomechanics. Pain may occur within the first two weeks post-stroke but typically emerges between one to three months afterwards [2]. Hemiplegic shoulder pain (HSP) significantly reduces patients’ functional abilities and rehabilitation potential, although effective pain management enhances participation in rehabilitation, improving functional capacity and quality of life [2].

Given that HSP can stem from various causes, a wide range of physical therapy approaches are employed, including conventional rehabilitation techniques, neuromuscular electrical nerve stimulation (NMES), transcutaneous electrical nerve stimulation (TENS), kinesio taping, slings, injections and acupuncture [3]. One of these treatment methods is light amplification by stimulated emission of radiation (laser), which utilizes intensified light. The principles of laser therapy are based on the quantum concept [4]. The basic working principle of low-level laser therapy (LLLT) involves photon energy emitted from a light source passing through specific tissue, thought to enhance local blood circulation, reduce inflammation and promote tissue healing. These mechanisms are particularly beneficial for post-stroke patients suffering from shoulder pain and dysfunction. Consequently, laser beams are employed in medicine for their regenerative, biostimulant, analgesic, anti-inflammatory and anti-edematous effects [4]. Previous studies have explored the use of laser therapy in conditions such as knee osteoarthritis [5] and shoulder adhesive capsulitis [6], as well as in hemiplegia [7].

Neuromuscular electrical nerve stimulation (NMES) induces muscle contractions using electrical pulses delivered to muscles through superficial electrodes. NMES mimics the action potential from the central nervous system, producing muscle contractions [8].

Patients with hemiplegic shoulder pain are generally enrolled in a conventional physical therapy program; however, we believe that adding LLLT to this program could lead to faster and more effective tissue healing due to its features such as increasing cell metabolism, enhancing blood circulation and reducing inflammation [4]. Additionally, incorporating NMES could further improve functionality and accelerate the recovery process by increasing muscle contraction and activating central stimulation [8]. To date, no study has compared LLLT and NMES for the treatment of hemiplegic shoulder pain. This study aimed to investigate whether the addition of LLLT or NMES to conventional physical therapy exercises in stroke patients with HSP provides additional benefits for pain, spasticity and upper extremity function, as well as to determine if one treatment modality is superior to the other.

## Materials and methods

This study was designed as a prospective randomized controlled trial. It included seventy-five hemiplegic patients aged 51 to 73 years, who had shoulder pain and were diagnosed with a first-time ischemic stroke. These patients applied to the Physical Therapy and Rehabilitation Outpatient Clinic between December 2023 and May 2024. Exclusion criteria were as follows: patients with other central nervous system diseases, those who received injections or physical therapy in the same shoulder in the last three months, those with a history of shoulder surgery, cervical radiculopathy, inflammatory rheumatic diseases or infections, serious cardiovascular diseases (such as heart failure, arrhythmia, or myocardial infarction) that would affect functional status, diseases causing cognitive dysfunction (such as Alzheimer’s disease or dementia), severe visual loss, malignancy and psychiatric diseases. All patients were informed prior to the study and an informed consent form was obtained. The study was conducted following the principles of the Declaration of Helsinki, as well as approved by the ethics committee of the University (2023 − 174) and registered in Clinical Trials NCT06428851.

The power analysis of the study was performed by the G power program, based on the study conducted by Korkmaz et al. (Power = 0.80; α = 0.05; d (effect size) = 0.70), with 25 patients in each group and 75 patients in three groups in total [7].

The patients included in the study were divided into three groups by the same physiotherapist by an envelope drawing method. All evaluations were performed by the same researcher who was blinded to the type of treatment. All patients underwent a conventional physical therapy program, five days a week for four weeks, for a total of twenty sessions. In this program, classical physical therapy exercises were applied to all patients according to the patient’s needs and neurologic level. These exercises were determined by the physiotherapist according to the functional status of the patient and consist of passive, passive assisted, active range of motion exercises, stretching and strengthening exercises, as well as mobilization exercises.

**Fig. 1:**
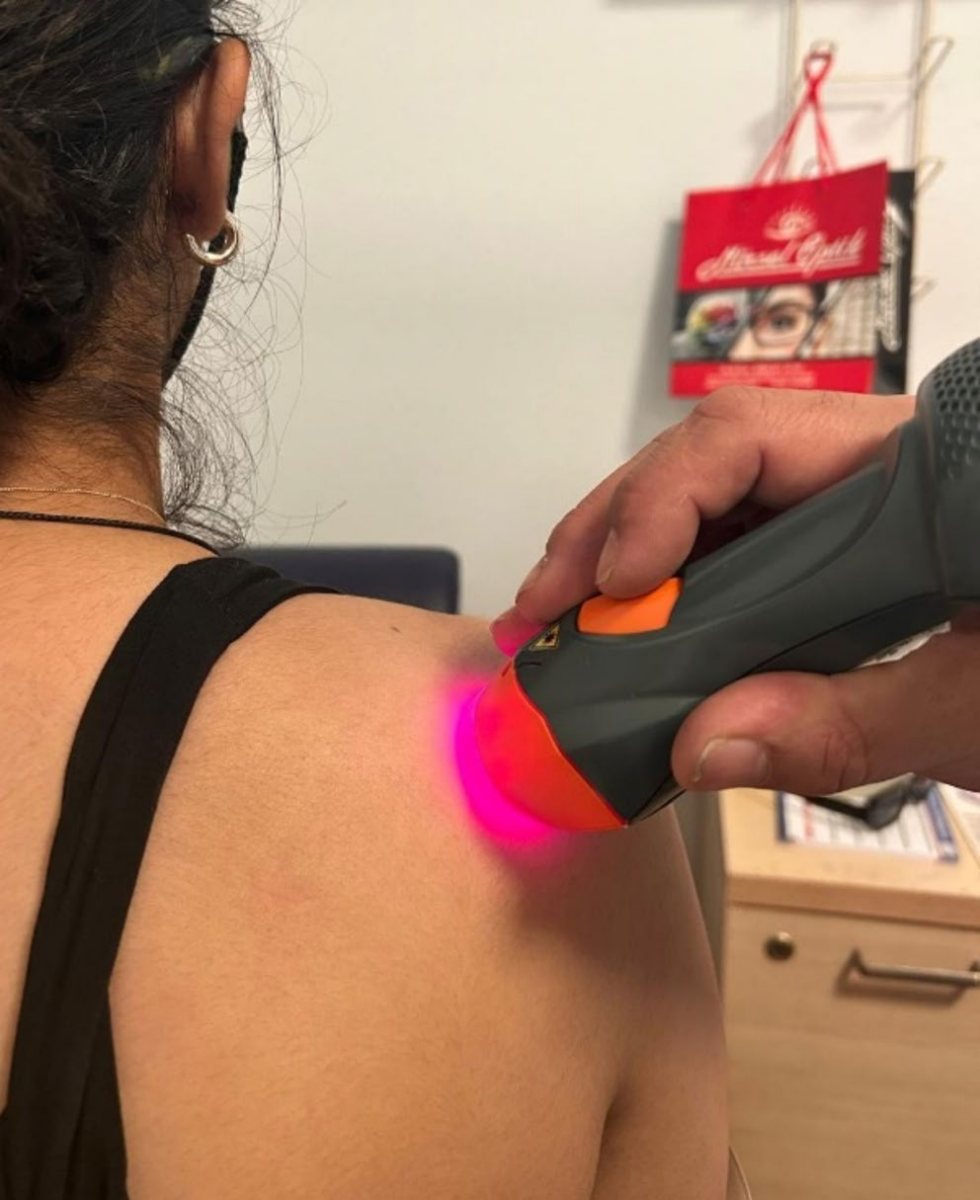
Application of LLLT

Group 1: The LLLT group (n:25) received laser treatment (Multiwave locked system MHPI 75) for three days a week for four weeks in addition to classical physical therapy. The device, equipped with a Multiwave Locked System, operates at a wavelength of 904 nm (in the invisible spectrum) with a power output of 100 mW and a spot size of 0.07 cm². A continuous laser mode was applied for 30 s at each of the nine points along the glenohumeral joint. Each point received irradiation at a power intensity of 3 J/cm², resulting in a total energy delivery of 27 J per treatment (Fig. 1). The parameters adhered to the World Association of Laser Therapy (WALT) recommendations for shoulder problems [9]. The laser device is calibrated annually by the same manufacturer on a regular basis.

**Fig. 2:**
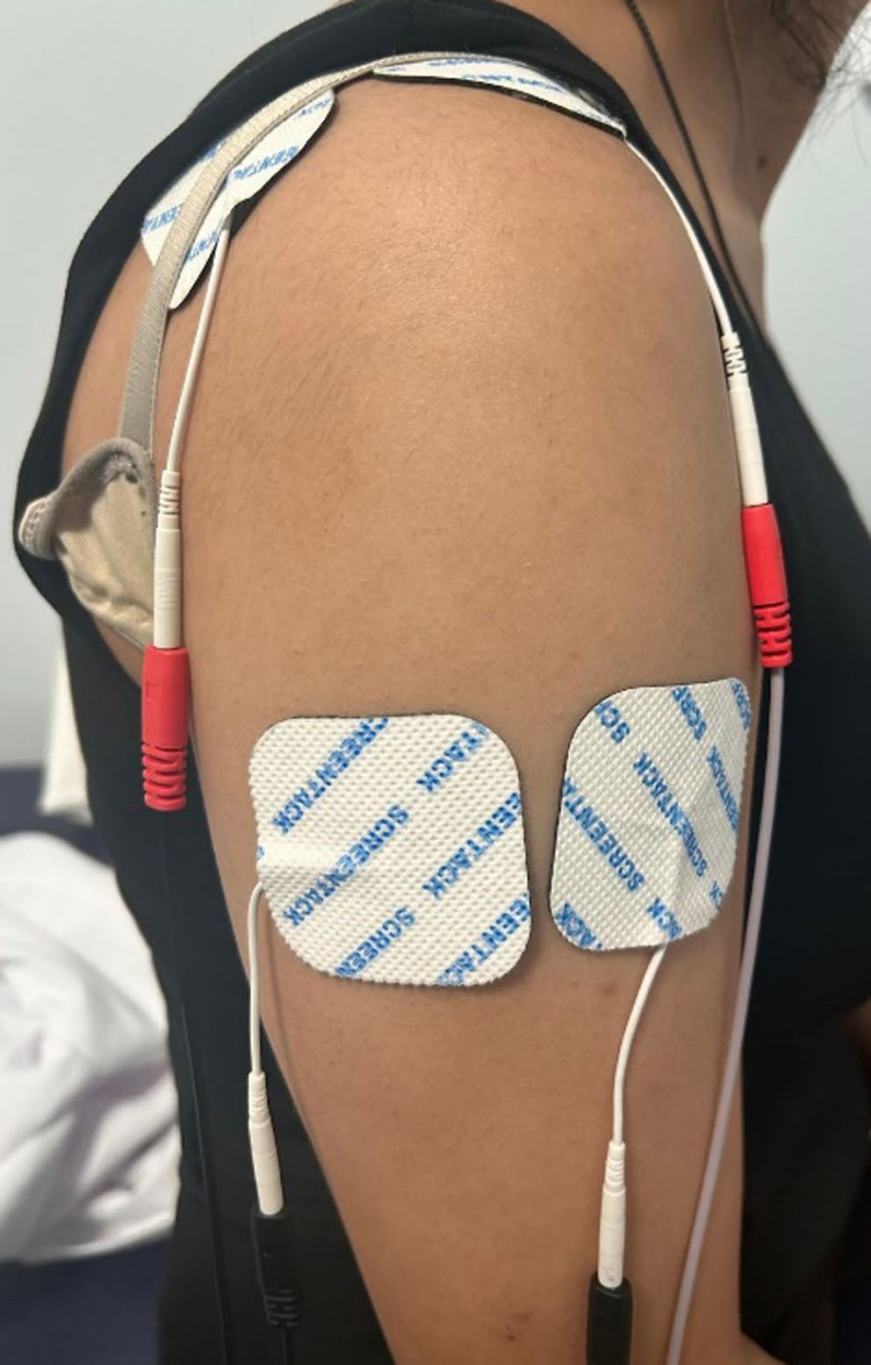
Application of NMES

Group 2: NMES group (n:25) (Chattanooga Intelect Advanced) received electrical stimulation of the paretic shoulder abductor muscles for twenty minutes, for five days a week for four weeks, using symmetrical biphasic rectangular waveforms with a frequency of 30 Hz, a pulse duration of 250 µs and a cycle of 2.5 s on and 2.5 s off, as recommended in previous literature [10, 11]. 4 self-adhesive surface electrodes, each measuring 5 × 5 cm were placed over the motor points near the middle of the supraspinatus and deltoid muscles (Fig. 2). These electrodes stimulate muscle movement the intensity of the electrical stimulation was meticulously adjusted to each patient’s maximum tolerance level (amplitude between 0 and 150 mA).

Group 3: Control group (n:25), conventional physical therapy program was applied.

Demographic characteristics of the patients (age, gender, stroke duration, marital status, dominant extremity, affected extremity) were recorded. The patients were evaluated twice, before enrolment and after the four-week physical therapy program. This evaluation noted stroke stages, spasticity levels, functional status and upper extremity functions.

The upper extremity functions of the patients were evaluated with Fugl-Meyer, stroke-specific performance-based scale and each parameter was scored between 0 and 2. A higher score indicates better performance [12].

The pain level of the patients was measured with a scale given to the patient; the visual analog scale (VAS). Patients rate their pain between 0 and 10. It consists of scores from 0 (no pain) to 10 (most severe pain) [13].

The Shoulder Pain and Disability Index (SPADI) was used for shoulder functional status; this is a patient-completed scale consisting of two parts, the first part SPADI pain includes five items related to pain and the second part SPADI disability includes eight items related to disability. High scores indicate high pain and disability [14].

The Barthel index, of which a Turkish adaptation of the scale was made [15], was filled by the responsible researcher after the examinations, to evaluate the activities of daily living of the patients. The Barthel index consists of ten items related to activities of daily living and mobility. The total score is between 0 à 100, 0 means fully dependent and 100 means fully independent [15].

The Brunnstrom Recovery Stage (BRS) is a six-step scale that includes movement patterns in which recovery gradually increases, to evaluate separately the upper extremity (UE), hand and lower extremity (LE). Higher values indicate better motor recovery [16].

To evaluate spasticity, the Modified Ashworth Scale (MAS), was used. This scale evaluates muscle tone between 0 normal and 4 being rigid [17].

### Statistical analysis

The statistical analyses were conducted using the SPSS for Windows, version 21.0. Both visual (histogram and probability graphs) and analytical (Kolmogorov-Smirnov/Shapiro-Wilk tests) methods were employed to assess whether the variables followed a normal distribution. For variables that did not conform to a normal distribution, descriptive statistics were presented using medians and interquartile ranges, while ordinal variables were summarized in frequency tables. The chi-square test was applied to compare categorical variables between groups. For non-parametric variables, the Mann-Whitney U test was utilized. Temporal changes in parameters, which did not adhere to a normal distribution, were examined for statistical significance using the Wilcoxon test. A type-1 error rate of 5% was considered for determining statistical significance.

## Results

Demographic characteristics of the groups are shown in Table 1. The groups were statistically similar in terms of age, gender, marital status, stroke duration, dominant arm, affected arm, BRS stages, MAS level, Bartel index, Fugl Meyer stage, SPADI pain, SPADI disability and VAS levels before treatment.

**Table 1:** Demographic and clinical characteristics of the groups

	LLLT Group *n* = 25	NMES Group *n* = 25	Control Group *n* = 25	p* value
Age (year)	58 (53-69.5)	60 (51.5–73)	65 (59.5–69.5)	0.402
Gender (female, %)	18 (72%)	10 (40%)	12 (48%)	0.062
Education level				0.176
0–8 years	13 (52%)	19 (76%)	20 (80%)	
9–12 years	4 (16%)	2 (8%)	3 (12%)	
> 12 years	8 (32%)	4 (16%)	2 (8%)	
Marital status (married %)	22 (88%)	23 (92%)	21 (84%)	0.685
Dominant arm (right %)	25 (100%)	25 (100%)	25 (100%)	
Affected arm (right %)	14 (56%)	13 (52%)	17 (68%)	0.489
Disease duration (month)	4 (2-9.5)	4 (2.5-7)	6 (3.5–11.5)	0.174
BRS Upper extremity pre	4 (3–5)	4 (2–5)	4 (3–5)	0.880
Upper extremity post	5 (4–5)	5 (4–5)	4 (3–5)	
	*p* = 0.001	*p* = 0.002	*p* = 0.025	
BRS Hand pre	4 (3.5-5)	4 (2–5)	4 (2–5)	0.642
Hand post	5 (4–5)	5 (3.5-5)	4(3–5)	
	*p* < 0.001	*P* = 0.001	*p* = 0.008	
MAS Shoulder pre	0 (0–2)	0 (0–0)	0 (0–0)	0.713
Shoulder post	0 (0–1)	0 (0–0)	0(0–0)	
	*p* = 0.008	*p* = 0.102	*p* = 0.157	
MAS Elbow pre	0 (0–2)	0 (0–2)	0 (0–1)	0.964
Elbow post	0 (0–1)	0 (0–0)	0 (0–1)	
	*p* = 0.008	*P* = 0.038	*p* = 0.083	
MAS Hand pre	0 (0–2)	0 (0-0.5)	0 (0–1)	0.847
Hand post	0 (0–1)	0 (0-0.5)	0 (0–1)	
	*p* = 0.008	*P* = 0.063	*p* = 0.083	
BARTEL pre	70 (25–90)	50 (25-87.5)	55 (25-82.5)	0.977
BARTEL post	85 (47.5–100)	70 (47.5–90)	60 (30-87.5)	
	*p* < 0.001	*p* < 0.001	*p* = 0.001	
FUGL MEYER pre	51 (33-64.5)	51 (32.5–66)	51 (33–66)	0.758
FUGL MEYER post	66 (50–66)	66 (40.5–66)	55 (33–66)	
	*p* < 0.001	*p* = 0.001	*p* = 0.012	
SPADI pain pre	25 (14-38.5)	24 (10–32)	23 (14.5–32)	0.558
pain post	10 (4-13.5)	8 (4.5–20.5)	20 (12–28)	
	*p* < 0.001	*p* < 0.001	*p* < 0.001	
SPADI disability pre	40 (9–73)	40 (7–76)	30 (4–75)	0.847
disability post	25 (3–44)	26 (4.5–48)	28 (1.5–72)	
	*p* < 0.001	*p* < 0.001	*p* < 0.001	
VAS pre	5 (4–6)	5 (4-5.5)	5 (3-6.5)	0.698
VAS post	2 (0,5 − 2)	2 (1–3)	4 (3-5.5)	
	*p* < 0.001	*p* < 0.001	*p* = 0.001	

p values were determined temporal changes of intragroups and p* values were determined the baseline differences between groups. LLLT: low-level laser therapy, NMES: neuromuscular electrical nerve stimulation, BRS: Brunnstrom recovery stage, MAS: modified Ashworth scale, SPADI: shoulder pain and disability index, VAS: visual analog scale, pre: pretreatment, post: posttreatment

When the groups were evaluated within themselves prior and after treatment, statistically significant improvements were found in BRS UE, BRS Hand, Barthel, SPADI pain, SPADI disability, Fugl Meyer and VAS values in all three groups. Shoulder, elbow and hand MAS scores decreased statistically significantly only in the laser group (p:0.008, p:0.008, p:0.008, p:0.008) (Table 1).

When the improvement in the groups was compared with each other, the groups were similar in terms of BRS UE and BRS hand. There was a statistically significant increase in Bartel and a statistically significant decrease in SPADI pain, SPADI disability and VAS values in the LLLT and NMES groups compared to the control group, but the LLLT and NMES groups were statistically similar (Table 2). No statistically significant difference was found between the groups in terms of MAS (Table 2).

**Table 2:** The intergroup differences of temporal changes

	*p* value between LLLT -NMES	*p* value between LLLT-Control	*p* value between NMES–Control	*p* value between groups
BRS upper extremity	0.722	0.023	0.050	0.057
BRS hand	0.887	0.075	0.064	0.117
MAS shoulder	0.959	0.615	0.589	0.840
MAS elbow	0.441	0.078	0.369	0.222
MAS hand	0.232	0.078	0.618	0.177
BARTEL	0.224	**< 0.001**	**0.017**	**< 0.001**
FUGL MEYER	0.342	**0.002**	0.035	**0.007**
SPADI pain	0.038	**< 0.001**	**< 0.001**	**< 0.001**
SPADI disability	0.892	**0.001**	**0.001**	**0.001**
VAS	0.114	**< 0.001**	**< 0.001**	**< 0.001**

p values in bold indicate statistical significance. LLLT: low-level laser therapy, NMES: neuromuscular electrical nerve stimulation, BRS: Brunnstrom recovery stage, MAS: modified Ashworth scale, SPADI: shoulder pain and disability index, VAS: visual analog scale

## Discussion

The study compared the effects of LLLT and NMES to conventional exercise therapy. Results indicated improvements in shoulder function, disability and pain across all three groups of hemiplegic patients. However, the LLLT and NMES groups experienced greater improvements compared to the control group, with similar outcomes between LLLT and NMES. Notably, a reduction in spasticity levels was observed only in the LLLT group.

HSP is a common and debilitating complication following a stroke, although, for different etiologies, it ultimately significantly affects the quality of life and functional independence of patients and may reduce patients’ potential for recovery.

High-intensity laser therapy (HILT) and LLLT have been studied extensively for their potential benefits in pain management and tissue repair for some different conditions such as knee pain [18], chronic low back pain [19], lateral epicondylitis [20] and shoulder pain [21]. There are also studies on the hemiplegic shoulder with laser [22], but to our knowledge, there is no study comparing LLLT and NMES in the literature. Research by Kheshie et al. [5] and Ordahan et al. [6] demonstrated the efficacy of both HILT and LLLT in reducing pain and improving function in musculoskeletal conditions, such as knee osteoarthritis and adhesive capsulitis. In the context of HSP, Korkmaz et al. [7] conducted a randomized controlled study showing that HILT significantly reduced pain and improved shoulder function in post-stroke patients. In another study of seventy patients, laser therapy was found to be more effective in reducing pain than TENS [23], similarly, in our study, both VAS and SPADI pain parameters decreased significantly in the LLLT and NMES group compared to the control group. The reason for this improvement can be partly explained by the distinct mechanisms of action of LLLT and NMES. LLLT primarily works by stimulating mitochondrial activity, which enhances ATP production and cellular repair processes, thus contributing to the reduction of inflammation and pain relief [4]. NMES, on the other hand, functions by eliciting muscle contractions, which enhance muscle strength, improve local circulation and potentially reduce the risk of disuse atrophy [10]. These effects are crucial for hemiplegic patients, as muscle weakness and atrophy significantly impact functional outcomes.

In the study by Lavi et al., NMES was found to be more effective than sham NMES in improving upper extremity functions and hand skills [8]. Similarly, Lin and colleagues applied NMES in addition to conventional physical therapy and found that NMES was more effective in enhancing upper extremity functions, with the effect persisting at the six-month follow-up after treatment [24]. In a study comparing HILT with conventional physical therapy, HILT was found to be more effective on upper extremity functions [25]. In our study, similar to these findings, upper extremity functions in the LLLT and NMES groups were found to be more effective than conventional physical therapy. However, there was no significant difference in shoulder pain and improvement of shoulder function between the LLLT and NMES groups. When comparing LLLT and NMES, the similar efficacy in terms of pain relief and functional improvement could be attributed to their complementary mechanisms. While LLLT primarily addresses pain through anti-inflammatory effects, NMES enhances muscle activation and functional use of the upper extremity, potentially leading to broader benefits in overall motor function. However, in our study, both interventions failed to show superiority over each other, which could be due to the limited follow-up period. As highlighted in previous studies, the long-term benefits of NMES are evident, suggesting that a longer follow-up might have provided further insights into the differences between these interventions [8, 24]. Also, it is important to note that the significant differences in patient selection criteria, types of lasers used, application parameters and treatment sites across the mentioned studies (including our own) make it challenging to draw reliable comparisons.

There are conflicting results regarding the effects of NMES on spasticity. While Zhou et al. found no reduction in spasticity with NMES treatment [26], a meta-analysis reported that most studies showed a decrease in spasticity [27]. In Zhou et al.’s study, nearly half of the patients had a hemorrhagic stroke, whereas all patients in our study had an ischemic stroke, which could account for the different results. Regarding laser therapy, there are few studies available. In one report, no difference in spasticity was found between the laser group and the control group [7]. In our study, although a reduction in shoulder spasticity was observed in the LLLT group, there was no significant difference between the LLLT, NMES and control groups and no reduction in spasticity was observed with NMES. This may be due to our short follow-up period and patient selection criteria, the latter being focused on the presence of pain rather than a specific underlying cause, due to the lack of consensus on the diagnosis, classification and treatment of shoulder pain.

When comparing LLLT and NMES, both modalities have shown promising results in managing HSP and enhancing upper extremity functions. Laser’s non-invasive nature and its ability to provide rapid pain relief make it an attractive option for many patients. However, the studies by Korkmaz et al. [7] and others suggest that while laser can be highly effective, its benefits may be limited to pain relief and immediate functional improvements. On the other hand, NMES offers a more comprehensive approach by not only alleviating pain but also improving muscle strength and preventing atrophy. The studies by Ada et al. [11] and Lin et al. [24] suggest that NMES offers improvements in motor recovery and functioning. However, there are few studies in the literature that compare LLLT and NMES. In a one study comparing them for knee osteoarthritis, functionality improved in both groups, but an increase in muscle strength and muscle mass was observed only in the NMES group [28]. Since assessing isolated muscle strength in the acute phase of hemiplegia is challenging, we did not evaluate this in our study. However, no difference was found between the groups regarding BRS motor stages.

Therefore, conducting studies that compare LLLT and NMES in HSP could provide more comprehensive results. And also clinicians should consider the specific needs and conditions of each patient when selecting the most appropriate intervention. Future research should focus on comparative studies with larger sample sizes and long-term follow-up to further elucidate the relative benefits of these therapies.

### Limitations

In the study, pathologies causing shoulder pain were not examined separately and patients were evaluated only at the beginning and end of treatment, so we are unable to comment on long-term outcomes. Due to the nature of the study, the practitioners were aware of the group assignments. Furthermore, for ethical reasons, the same exercise program was applied to all patients and there were no sham NMES or sham LLLT groups. Subacute and chronic hemiplegic patients were included in the study, as HSP is more common in these phases. No statistically significant difference was found in the duration of the disease among the patients included in the study; however, naturally, the rate of recovery slows in the later weeks, which could influence the study’s results.

## Conclusion

LLLT, NMES and conventional exercise therapy have all shown efficacy in treating HSP, improving upper extremity functions and reducing disability. LLLT and NMES were more effective than conventional exercise, with similar effects between LLLT and NMES.
